# High-Throughput Single-Cell Proteomics of *In Vivo* Cells

**DOI:** 10.1016/j.mcpro.2025.101018

**Published:** 2025-06-20

**Authors:** Shiri Karagach, Joachim Smollich, Ofir Atrakchi, Vishnu Mohan, Tamar Geiger

**Affiliations:** Department of Molecular Cell Biology, Weizmann Institute of Science, Rehovot, Israel

**Keywords:** single-cell proteomics, high-throughput, mass spectrometry, automated sample preparation, tumor macrophages, slice-PASEF, cell fixation, 1536-well plates, murine model, cancer research, lung metastasis

## Abstract

Single-cell mass spectrometry-based proteomics (SCP) can resolve cellular heterogeneity in complex biological systems and provide a system-level view of the proteome of each cell. Major advancements in SCP methodologies have been introduced in recent years, providing highly sensitive sample preparation methods and mass spectrometric technologies. However, most studies present limited throughput and mainly focus on the analysis of cultured cells. To enhance the depth, accuracy, and throughput of SCP for tumor analysis, we developed an automated, high-throughput pipeline that enables the analysis of 1536 single cells in a single experiment. This approach integrates low-volume sample preparation, automated sample purification, and LC-MS analysis with the Slice-PASEF method. Integration of these methodologies into a streamlined pipeline led to a robust and reproducible identification of more than 3000 proteins per cell. We applied this pipeline to analyze tumor macrophages in a murine lung metastasis model. We identified over 1700 proteins per cell, including key macrophage markers and more than 500 differentially expressed proteins between tumor and control macrophages. PCA analysis successfully separated these populations, revealing the utility of SCP in capturing biologically relevant signals in the tumor microenvironment. Our results demonstrate a robust and scalable pipeline poised to advance single-cell proteomics in cancer research.

## Highlights

• We present one of the first single-cell proteomics studies applied to an *in vivo* murine cancer model.
• We have established a semi-automated workflow from the cell isolation to the Evotip loading, including a new protocol for 1536-well plates.
• We present a high-throughput SCP method, which is critical, not only for achieving statistically significant results but also for capturing biological heterogeneity and identifying potential cell subpopulations.
• We optimized protocols for cell fixation to preserve the proteome and provide a pause point in the long protocols.

## Graphical Abstract

**Figure:**
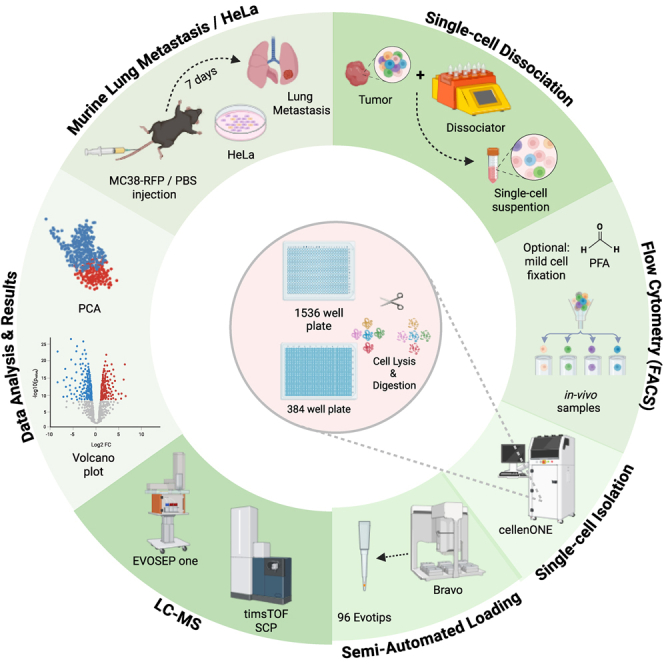

## In Brief

We developed an automated, high-throughput single-cell proteomics pipeline capable of analyzing 1536 single cells per experiment using low-volume sample prep, automated purification, and LC-MS analysis. Applied to tumor macrophages in a murine lung metastasis model, the method identified over 1700 proteins per cell and revealed distinct proteomic profiles between tumor and control macrophages, demonstrating its robustness and potential for advancing cancer single-cell proteomics.

Analyzing biological systems at the single-cell level offers the advantage of capturing pure cellular signals, eliminating the need to average signals across predefined cell populations based on known markers. The advent of single-cell RNA sequencing (scRNA-seq) has revolutionized our ability to identify novel cell populations and trace cellular trajectories, significantly advancing our understanding of developmental processes and dynamic cellular changes across various biological systems. In cancer research, scRNA-seq has been instrumental in identifying unique cell populations that may influence therapeutic responses—insights often masked in population-level analyses due to the averaging of signals, which obscures the presence of rare cell types (1, 2).

Despite its transformative impact, scRNA-seq has limitations. RNA-level analyses alone cannot fully capture cellular function, as RNA and protein expression are not always correlated (3, 4). Numerous studies, including ours, highlight the importance of protein-level analyses for understanding cellular pathways (3, 5). Pathways involving complex molecular machinery, such as the ribosome, spliceosome, electron transport chain, and proteasomes, often display low RNA-protein correlations. In contrast, pathways under transcriptional regulation, such as the interferon pathway, exhibit high RNA–protein correlations. Signaling, metabolic, and adhesion pathways typically show moderate correlations (around 0.5), though these correlations vary widely across biological systems (6, 7, 8, 9). Additionally, scRNA-seq is often limited by low transcriptional coverage per cell, high variability in signal between cells, and significant amounts of missing data (10). As a result, rigorous statistical analyses generally require data from thousands of cells to define well-resolved cellular clusters with sufficient depth.

The recognition of scRNA-seq's capabilities and limitations has spurred the development of single-cell proteomics (SCP) technologies to broaden the scope of mass spectrometric analyses. Advances in mass spectrometry sensitivity, driven by improved ion transfer efficiency and reduced background noise, now permit the analysis of sub-nanogram protein quantities (11, 12, 13, 14, 15, 16, 17). Enhanced sample preparation techniques, such as minimizing protein absorption to plasticware and reducing sample volumes, improve protein recovery and digestion efficiency by increasing protein concentration (18, 19, 20, 21). Efforts were made to improve single-cell throughput during sample preparation by incorporating nanowell chips, glass slides, or plates (16, 22, 23). To date, most SCP studies have focused on single cultured cells, demonstrating advancements in each of these parameters. However, analyzing *in vivo* tumor cells presents unique challenges due to their inherent complexity, including variability in cell size, cell type, and the presence of cell debris, which can complicate sample processing and data interpretation. In this study, we aim to further advance SCP technology for the analysis of tumor cells. To this end, we developed an automated sample preparation protocol for high-throughput single-cell processing using 1536-well plates. By incorporating cell fixation, we established a robust and scalable pipeline capable of overcoming the technical hurdles of single-cell tumor proteomics, paving the way for more reliable and comprehensive analyses of these heterogeneous cell populations.

## Experimental Procedures

### Cell Culture

HeLa cells, MC38-RFP murine colorectal cancer cells and RAW 264.7 murine macrophage cells were cultured in DMEM supplemented with 10% FBS and 1% Penicillin-Streptomycin at 37 °C with 5% CO_2_. To generate single-cell suspensions, cells were dissociated from the culture plates with trypsin, washed with phosphate-buffered saline (PBS), and diluted to a concentration of 100 to 200 cells/μl in degassed PBS for downstream single-cell separation. In the fixation experiments, HeLa cells were resuspended in 1.5 ml of paraformaldehyde (PFA) dissolved in PBS to three concentrations: 0.5%, 2%, and 4%. The samples were incubated at room temperature for three time periods: 5, 15, and 30 min. Non-fixed control samples were incubated in PBS alone. Following incubation, the cells were centrifuged at 300 rpm for 5 min, washed once with PBS, and resuspended in FACS buffer (1× PBS with 1% FCS and 1 mM EDTA). Half of the samples were analyzed on the same day, while the remaining half were stored at 4 °C overnight for later analysis.

### Mouse Models and Macrophage Isolation

MC38 colorectal cancer cells were injected into the tail vein of 10-week-old C57BL6 mice (Harlan). Mice were injected with 10^6^ cells in 100 μl PBS or with the same volume of PBS as a control. One week post-injection, mice were euthanized, and lungs were removed and dissociated into single cells using the lung dissociation kit (Miltenyi) on the gentleMACS Dissociator (Miltenyi). Red blood cells were removed using the red blood cell lysis buffer. Cells were then stained with Live/Dead Fixable Blue (L34961, Thermo Fisher Scientific) to remove dead cells and subsequently labeled with antibodies for macrophage sorting using the following gating strategy: CD45^+^ (BioLegend #103107), CD11b intermediate (BD Biosciences #740861), CD11c^+^ (BD Biosciences #565452), and CD64^+^ (BioLegend #139332). The isolation of pooled macrophages was performed by flow cytometry on the FACS-Aria III sorter (BD-Biosciences), followed by single-cell dispensing using the cellenONE instrument. All animal experiments were approved by the Institutional Animal Care and Use Committee, application #00030123.

### Single-Cell Dispensing on cellenONE and In-Solution Trypsin Digestion

Single cells were isolated using the cellenONE automated image-based single-cell dispenser. This instrument utilizes piezoelectric technology for gentle, picoliter-volume acoustic dispensing, which enhances cell viability and allows for the miniaturization of sample preparation workflows. Uncoated Piezo Dispensing Capillaries (PDCs) of size M or L were used in all experiments. The lysis and digestion buffer MasterMix included 0.2% n-dodecyl-β-D-maltoside (DDM), 20 ng/μl Sequencing Grade Modified Trypsin (Promega), and 100 mM Triethylammonium bicarbonate (TEAB) buffer. A polystyrene 384-well plate served as the probe, and polypropylene 384-well plates (Eppendorf) or 1536-well plates (Greiner) were used as the target. To ensure droplet stability, the intermediate task DipDropCheck was performed every 14 wells during MasterMix dispensing. Relative humidity was maintained at 50%, and the temperature was regulated using a DewPoint set point to prevent condensation. After dispensing, the plates were covered and centrifuged for 1 min at 800*g*. The single-cell suspensions were prepared from tissue-cultured HeLa cells or dissociated mouse lungs, diluted into a final solution of 100 to 200 cells/μl suspension in degassed PBS. To ensure homogeneous sampling during the isolation process, the Mix&Take task was used to prevent cell settling. Based on cell mapping prior to isolation, diameters of 20 to 35 μm and 15 to 30 μm were used for HeLa cells and murine macrophages, respectively. A maximum elongation of 1.85 was applied to avoid the isolation of doublets or non-single cells. During isolation, humidity and temperature conditions matched those described for MasterMix dispensing. The plates were covered and centrifuged again for 1 min at 800*g*. Two different incubation approaches were tested: (1) Covered plate with an aluminum seal and (2) Water rehydration at 500 Hz. In both approaches, the plates were incubated at 50 °C for 2 h with 85% humidity to prevent evaporation. For the 1536-well plate, an in-house produced aluminum adaptor with an anti-corrosion coating was used to improve heat conductivity. After incubation, the plates were cooled to 20 °C and centrifuged again for 1 min at 800*g*. Automated dilution was performed using 5 μl of solvent A (99.9% water + 0.1% formic acid) with the Bravo liquid handler (Agilent). Finally, the plates were frozen at −80 °C or used for semi-automated Evotip loading.

### Semi-automated Evotip Loading

Sample clean-up and loading were performed based on the semi-automated Evotip loading protocol of Evosep for the Bravo liquid handler (Agilent). The publicly available protocol was adapted to the 96-ST head. For the semi-automated sample loading from 1536-well plates, the V10 10 μl tips (Agilent) were used. To avoid solvent leakage during solvent pipetting on the robot, “air gaps” (post aspiration volumes of 5 μl) were added to each pipetting step. In addition, to ensure complete pipetting without any sample loss, “blow-out” options were chosen when sample solutions were dispensed.

### LC-MS Analysis

LC-MS analyses were performed on the Evosep1 liquid chromatography system (Evosep) coupled to the timsTOF SCP mass spectrometer (Bruker). Peptides were separated on the Aurora Elite CSI 15 × 75 C18 UHPLC column (Ionopticks) using the Whisper Zoom 40 SPD method. Mass spectrometric acquisition was performed in a data-independent (dia) PASEF mode. For the diaPASEF mode, the ion accumulation and ramp time were set to 100 ms, and precursors in an ion mobility range from 1/K_0_ = 1.45 Vs cm^-2^ to 0.64 Vs cm^-2^ were analyzed over a mass to charge (m/z) range from 100 to 1700. Precursor ions for MS/MS analysis were isolated based on diaPASEF window placement covering an ion mobility range from 1/K_0_ = 0.75 Vs cm^-2^ to 1.2 Vs cm^-2^ and a mass to charge (m/z) range of 400 to 1000. The estimated cycle time was 0.64 s with 1 MS ramp and 5 MS/MS ramps, resulting in 75 MS/MS windows. The collision energy was ramped from 20 eV at 1/K_0_ = 0.6 Vs cm^-2^ to 59 eV at 1/K0 = 1.6 Vs cm^-2^. For the pydiAID-based sample acquisition, precursor ions for MS/MS analysis were isolated based on diaPASEF window placement covering an ion mobility range from 1/K_0_ = 0.7 Vs cm^-2^ to 1.4 Vs cm^-2^ and a mass to charge (m/z) range of 343.2 to 1393.7. PydiAID window details are provided in Supplemental Table S1. The estimated cycle time was 0.94 s with 1 MS ramp and 8 MS/MS ramps, resulting in 16 MS/MS windows. Singly charged precursors were excluded with a polygon filter. We used the publicly available Slice-PASEF window scheme for design (24). Slice-PASEF window details are provided in Supplemental Table S1. The method file “diaParameters_slice” was downloaded and included in the timsTOF SCP acquisition method file. For the generation of the pydiaAID window scheme we used the “py_diAID” open-source Python package.

### Data Analysis

DIA-NN version 1.9.1 (HeLa samples) and DIA-NN version 1.9.2 (murine macrophage samples and RAW 264.7 cell line samples) were used to analyze the mass spectrometric raw files. Analyses of HeLa files were performed using a library-based approach (spectral library provided by BRUKER containing 67,686 proteins and 13,827 genes). Library precursors were reannotated using a FASTA database (FASTA files UP000005640_9606.fasta and UP000005640_9606_additional.fasta, version August 2022). For the analysis of the murine macrophage samples, we used an in-house generated spectral library based on murine bulk proteomics samples (containing 29,438 proteins and 10,517 genes). Library precursors were reannotated using a FASTA database (FASTA files UP000000589_10090.fasta and UP000000589_10090_additional.fasta, version August 2022). We included 12 separately processed RAW 264.7 murine macrophage single cell samples in the same DIA-NN search as the primary macrophage single cell samples, to increase proteome coverage.

Trypsin was selected as a protease, with a maximum of one missed cleavage. DIA-NN default settings were used with the following exceptions: Precursors' charge range was set to 2 to 10. The “Unrelated runs” option was selected to determine optimal MS1 and MS2 accuracies and scan window range for each run individually. The precursor m/z range was set to 300 to 1700, and the "Fragment ion m/z range” was set to 200 to 1700. “C carbamidomethylation” modification was disabled. For Slice-PASEF samples, the “tims-scan” option was added as a command in the “Additional options” window. To generate annotated spectra, the “report-lib-info” command was enabled, ensuring that the DIA-NN “main report” contains comprehensive spectral library details alongside the standard quantification and identification results.

Protein group intensities were filtered at 1% false discovery rate (FDR) using a global q-value and a run-specific protein-level FDR threshold of 5% (“pg-matrix”). Precursor intensities were filtered at 1% FDR based on both a global and a run-specific q-value (“pr-matrix”). Data analysis was performed on the DIA-NN output tables “pg-matrix”, “pr-matrix”, and the “main report”.

To generate annotated spectra for single-hit proteins, the DIA-NN “main report” was used. Single-peptide proteins were identified by counting the number of unique peptides assigned to each protein, using the "Stripped.Sequence" entries from the “pr-matrix” (filtered at 1% FDR) as peptide sequences. For each single-hit protein, the corresponding peptide sequence and fragment information (fragment type [b-ion, y-ion], intensity, and m/z value) were extracted from the “main report” and matched to the spectral library data. Annotated spectra are of single-peptide proteins are provided in PRIDE PXD058457. (provided as PDF files in the Supplemental Material). The R scripts used for this analysis are included as commented code in the Supplemental Materials.

### Experimental Design and Statistical Rationale

All experiments analyzed single cells, which form biological replicates. All technical analyses of HeLa cells included at least 12 cells per group. These experiments were repeated at least three times, reaching similar conclusions. One representative experiment is presented in each graph. Each figure shows a separate experiment with no overlapping samples. *In vivo* tumor mouse analyses were performed on six mice, with more than 70 cells analyzed per group. The computational pipeline filtered single-cell samples to retain those with a minimum of 1000 protein IDs per cell, followed by the exclusion of three outlier single cells. Proteins detected in at least 30% of the samples were retained, resulting in a final dataset of 1573 protein IDs. Prior to principal component analysis (PCA), protein intensities were scaled using z-scores, and missing values were imputed based on a normal distribution with a downshift of 1.8 standard deviations and a width of 0.3 of the overall data distribution of the sample. To address batch effects arising from experiments conducted at different time points, the removeBatchEffect function from the ‘limma’ package was applied.

## Results

**Fig. 1:**
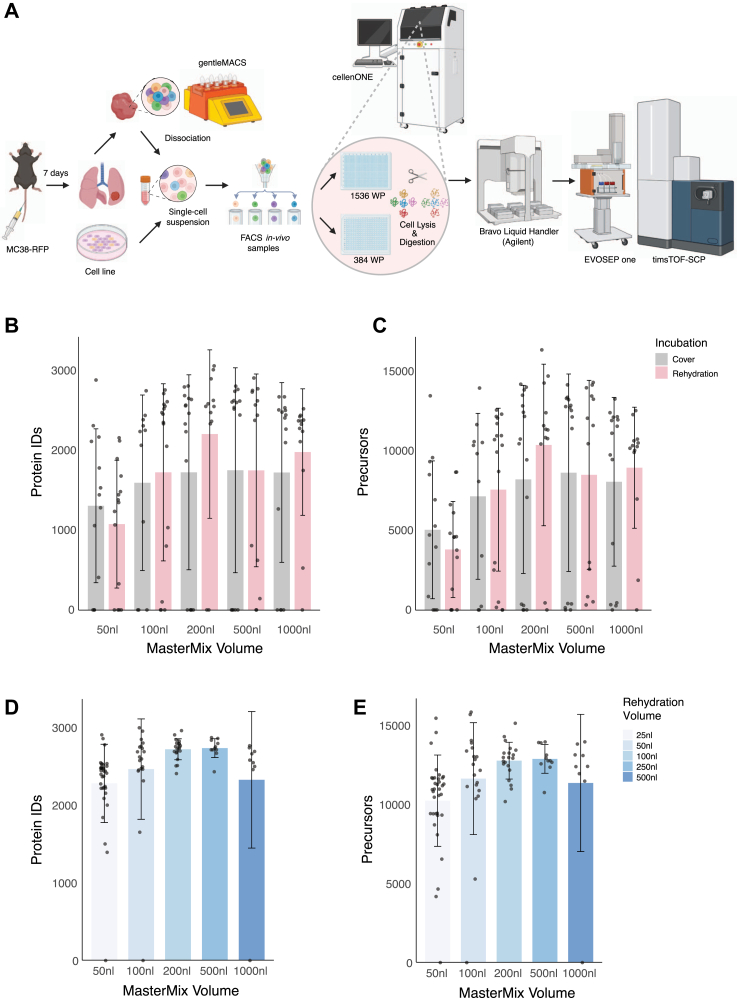
**Miniaturization of the SCP workflow.***A*, a schematic representation of the SCP workflow. Sample preparation is initiated with cultured cells or *in vivo* tumor samples. For murine samples, macrophages are pre-sorted using FACS and pooled prior to single-cell isolation. Single-cell suspensions are separated into single wells on the cellenONE dispenser directly into MasterMix in 384 or 1536 well-plates. Reactions are loaded onto Evotips on the Bravo liquid handler before LC-MS analysis on the Evosep1 coupled to the timsTOF SCP mass spectrometer. The figure is created with BioRender.com. *B*, bar plot shows the number of identified protein groups using increasing MasterMix volumes, comparing covered plates vs. rehydration with 300 nl of water. *C*, plot of the number of precursors for the same experiment as (*B*). *D*, same as (*B*), but with rehydration with volumes equal to 50% of the initial reaction volume. *E*, plot of the number of precursors of the same experiment as (*D*).

The application of single-cell proteomics to tumor samples demands robustness, depth, accuracy, and throughput. Building on previous advancements in low-volume sample preparation, we developed an experimental pipeline that integrates automated, high-throughput sample preparation for LC-MS analysis to achieve deep proteomic coverage (Fig. 1*A*). Our objective was to enable the analysis of over 1000 cells within a single experiment, a capability essential for *in vivo* studies and the foundation for advancing single-cell biological research.

### High-Throughput Single-Cell Proteomics Sample Preparation

As the initial step in sample preparation optimization, we developed a protocol for single-cell isolation on the cellenONE dispenser and in-solution trypsin digestion. The cellenONE system allows low-volume dispensing and reduces sample evaporation through controlled temperature and humidity. Additionally, it supports protocol execution in open plates while continuously rehydrating the wells. Following standard protocols, we assessed proteomic coverage by preparing samples in increasing volumes of MasterMix within a 384-well plate. In the first set of experiments, plates were covered to minimize evaporation. In the second set, we rehydrated each well with 300 nl of water during a 2-h digestion. Samples were subsequently loaded onto Evotips and analyzed on the timsTOF SCP. Our analyses found a consistent improvement in protein identification when using the rehydration protocol compared to covered wells (Fig. 1, *B* and *C*, Supplemental Table S1). Furthermore, we observed that reducing the sample volume below 100 nl significantly reduced proteomic depth, likely due to increased evaporation. Notably, proteome coverage showed only minor differences upon dehydration, with mean coverage ranging from 1800 to 2000 proteins and maximal coverage between 2900 and 3100 proteins, independent of the initial MasterMix volume. We speculated that the large 300 nl rehydration volume may have masked initial volume differences. We then repeated the experiments with rehydration volumes proportional to the initial reaction volumes. Specifically, rehydration with 50% of the initial reaction volume improved coverage, particularly for smaller reaction volumes of 50, 100, and 200 nl. Reactions using 50 nl were suboptimal, but reaction volumes of 100 to 200 nl, combined with rehydration of 50 to 100 nl, provided optimal results in a 384-well plate format (Fig. 1, *D* and *E*, Supplemental Table S1).

**Fig. 2:**
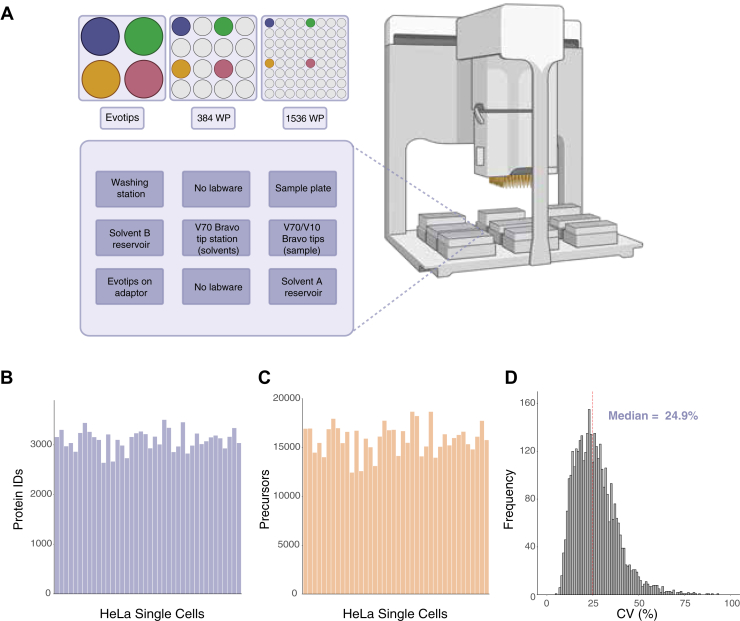
**Semi-automated Evotip loading.***A*, semi-automated Evotip loading protocol on the Bravo liquid handler enables sample loading from 384-well and 1536-well plates onto 96-well format Evotips. *B* and *C*, automated loading of 40 HeLa cells results in reproducible protein (*B*) and precursor (*C*) identification in 384 well-plates. *D*, determination of the coefficient of variation of each protein shows overall low CVs across 40 samples.

We automated the Evotip loading protocol on a Bravo liquid handler (Agilent) to further increase throughput and reproducibility. This protocol allows for the automated loading of 96 Evotips from 384-well or 1536-well plates while minimizing sample loss (Fig. 2*A*). A comparison of 40 single HeLa cells showed low variation in the number of peptides and proteins identified per cell (mean protein count 3101 ± 203 standard deviation, Fig. 2, *B* and *C*, Supplemental Table S2). Determination of the coefficient of variation (CV) for all detected proteins across all measured single cells showed a median CV of 24.9%, indicating high quantitative accuracy and robustness (Fig. 2*D*).

**Fig. 3:**
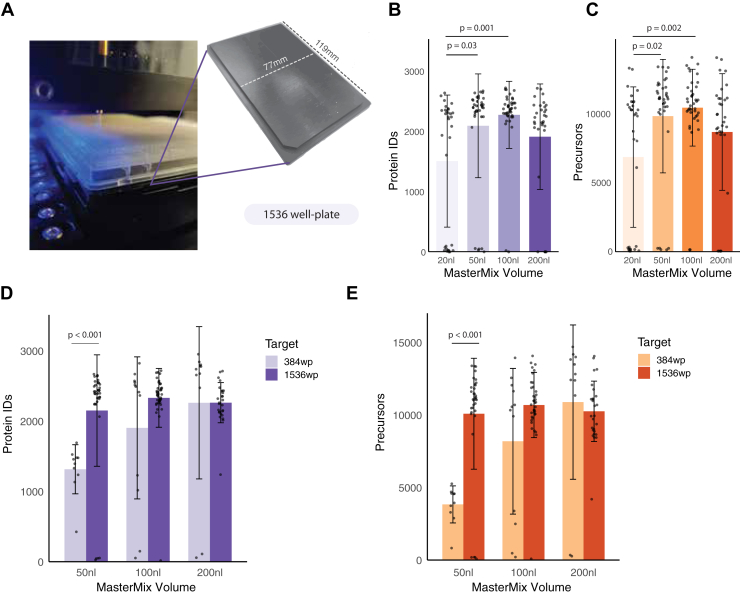
**SCP on 1536-well plate format.***A*, we designed and produced an aluminum adapter for heat conduction in the cellenONE dispenser. The Piezo Dispensing Capillary (PDC) is located right above the well to ensure droplet entry into the wells. *B* and *C*, optimization of sample volume shows the highest number of protein groups (*B*) and precursors (*C*) when using 100 nl MasterMix volume. *D* and *E*, a comparison between 384-well to 1536-well formats shows improved proteome (*D*) and precursor (*E*) coverage in 1536-well plates when using 50 to 100 nl reactions and similar results when using 200 nl volumes.

With the automated pipeline in place, we explored options to increase further sample preparation throughput—critical for single-cell proteomics in tumor analysis, where thousands of cells must be analyzed to define cellular clusters in an untargeted manner. To achieve this, we transitioned from 384-well to 1536-well plates, allowing reduced sample volumes while limiting rehydration due to the small well size. To accommodate the cellenONE system for this format, we designed a metal adaptor that fits the height of a 1536-well plate and facilitates temperature control during sample preparation (Fig. 3*A*). Testing MasterMix volumes ranging from 20 to 200 nl, we observed the highest peptide and protein identification rates with 100 nl volumes (Fig. 3, *B* and *C*, Supplemental Table S3). Direct comparisons between the two plate formats showed significantly greater proteome coverage in 1536-well plates versus 384-well plates for 50 to 100 nl reaction volumes (Fig. 3, *D* and *E*). Thus, we propose that optimal reaction volumes are 200 nl for 384-well plates and 100 nl for 1536-well plates. We recommend the 1536-well format for complex tissue samples to process large cell numbers simultaneously, minimizing potential proteomic changes due to delays in cell dispensing and reducing batch effects.

### Development of a Fixation Protocol for Single-Cell Proteomics

**Fig. 4:**
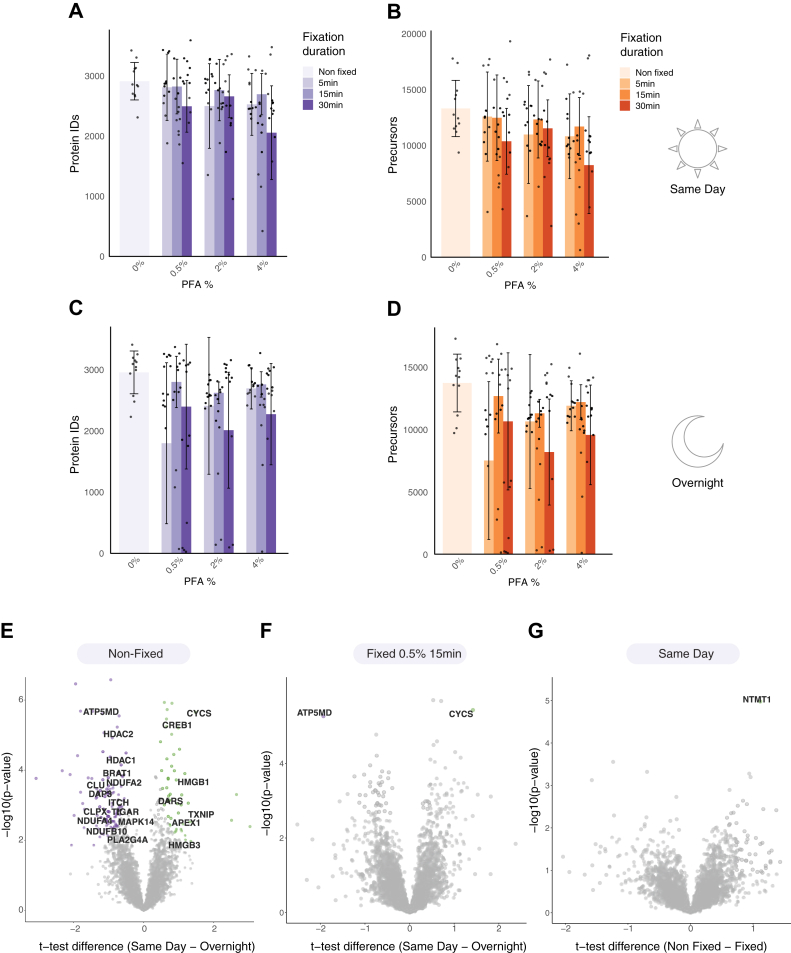
**SCP of fixed cells.** HeLa cells were fixed with increasing concentrations of PFA and for increasing incubation times. *A* and *B*, protein (*A*) and precursor (*B*) identification when cells were dispensed and lysed on the same day, immediately after fixation. *C* and *D*, protein (*C*) and precursor (*D*) identification when cells were dispensed after an overnight incubation. *E*, volcano plot shows the significantly changing proteins in non-fixed cells, between cells before and after an overnight incubation. *F*, volcano plot shows two significantly changing proteins in fixed cells between cells before (same day) and after overnight incubation at 4 °C. Non-significant proteins are depicted in *gray*. *G*, volcano plot shows no significantly changing proteins between fixed and non-fixed cells before an overnight incubation. *E–G*, two-sided T-tests were conducted with a permutation-based FDR cutoff of 5% and S_0_ = 0.1.

Cell fixation can alleviate the negative impact of long gaps between the cell suspension and lysis. Delayed cell lysis may lead to protein degradation and activation of stress signals that do not reflect the true biological state of the cells before cell suspension. Previous proteomic studies of archived tissue samples showed that formalin and formaldehyde have a minor impact on the proteins (25, 26), thereby allowing for robust analysis of fixed cells. To optimize a protocol for single-cell fixation, we compared the proteomic results obtained with increasing concentration and incubation time of PFA and compared them to non-fixed cells. Initial testing of cells taken immediately after fixation showed only a minor reduction in protein identification from HeLa cells fixed with 0.5% PFA for 5 and 15 min (Fig. 4, *A* and *B*, Supplemental Table S4). Higher concentrations and longer incubation times already decreased protein identification, presumably due to over-fixation and protein cross-linking. We then examined protein identification after overnight incubation. Adding a pause point after tissue disaggregation and before single cell dispensing may be required in lengthy sample preparation procedures, and therefore, it is required to ensure that cells and proteins are retained minimally altered. We incubated cells with PFA for varying durations, washed them with PBS, and kept them overnight at 4 °C. Like the same-day measurements, we found that 0.5% PFA incubation for 15 min resulted in only a minor reduction in protein identification from single cells (Fig. 4, *C* and *D*, Supplemental Table S4). Surprisingly, we found no reduction in protein identification in non-fixed cells despite the overnight incubation.

To deepen our investigation of proteomic changes upon fixation, we compared the proteomes of non-fixed single HeLa cells with and without an overnight incubation at 4 °C. We found 126 significantly changing proteins upon incubation overnight (two-sided Student’s *t* test FDR = 5%, S_0_ = 0.1; Fig. 4*E*; Supplemental Fig. S1), suggesting that fixation is useful to retain the proteomic state. The overnight incubation of non-fixed HeLa cells at 4 °C resulted in the upregulation of proteins involved in energy metabolism and stress, and cell cycle regulation. These include components of the oxidative phosphorylation (OXPHOS) pathway, including NDUFA4, NDUFA2, and NDUFA11, mitochondrial protease DEG1, and TIMM23, which facilitates mitochondrial protein import (Supplemental Fig. S1*A*). Additionally, cell cycle proteins, primarily G2/M-related proteins, were upregulated in these conditions. These include core mitotic regulators such as CDCA8 and AURKB, the spindle assembly checkpoint protein BUB1B, and the G2/M transition driver CCNB2. Previous studies show that cellular stress is often associated with G2/M arrest and higher mitochondrial protein content (27, 28). Therefore, we speculate that the stress conditions of cells stored in suspension for long durations might impact these processes, among others. Among the proteins that were downregulated upon overnight incubation, we found a network of proteins related to gene expression regulation, including epigenetic factors, splicing machinery, translation-related proteins, and so on. (Supplemental Fig. S1*B*). These results suggest that overnight incubation might impact gene expression patterns. We speculate that with longer suspension durations these protein changes might lead to cell death. In contrast, when comparing the proteomes before and after an overnight incubation, and of fixed to non-fixed cells on the same day, we found only 1 to 2 significantly changing proteins (Fig. 4, *F* and *G*). Therefore, we conclude that mild fixation retains the protein levels while only slightly affecting overall coverage. This way, fixation allows the experiments to pause without impacting biological results.

### Optimization of LC-MS Acquisition Methods

**Fig. 5:**
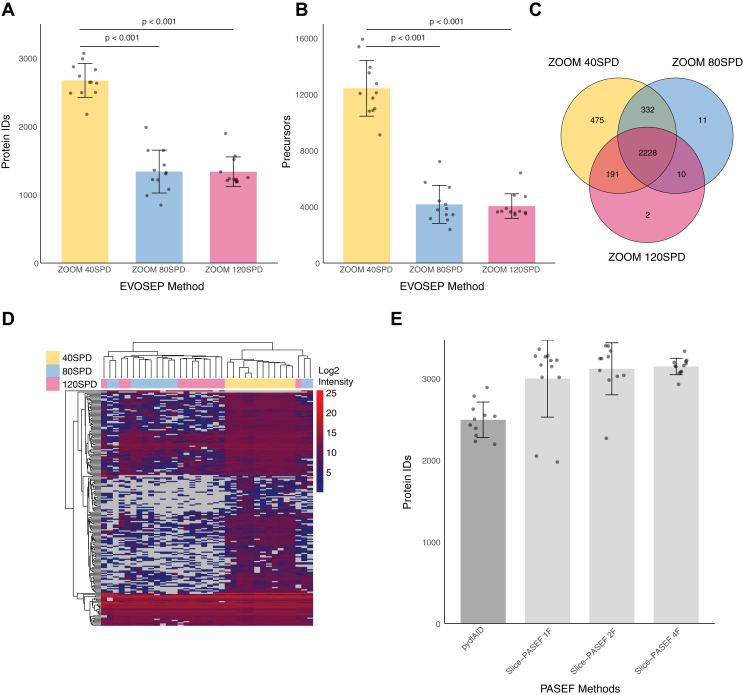
**Optimization of LC-MS parameters for SCP.***A* and *B*, a Comparison of three Evosep1 LC methods determines the number of proteins (*A*) and precursors (*B*) identified using 40, 80, and 120 SPD Whisper Zoom methods. *C*, a Venn diagram shows the protein overlap of analyses of 36 cells analyzed by 40, 80, and 120 SPD methods. *D*, hierarchical clustering of samples analyzed by each LC method shows similar expression levels of highly expressed proteins, but sporadic and inconsistent expression of lowly expressed proteins in 80 and 120 SPD, relative to 40 SPD. *E*, examination of Slice-PASEF shows a significant advantage compared to the pydiAID optimized method. A minimum protein ID cutoff was set to 1000 IDs.

The sample preparation procedures described above provide the pipeline to prepare 1536 samples in 1 to 2 days. Next, we examined the ability to improve LC-MS acquisition. Our experimental setup used the Evosep1 liquid chromatography system, which provides reproducible and accurate LC parameters. We used the Whisper Zoom 40 samples per day (SPD) method. To increase the throughput, we also tested shorter methods for the acquisition of 80 and 120 samples per day (80 SPD and 120 SPD) but found a marked reduction (>50%) in protein and peptide coverage (Fig. 5, *A* and *B*, Supplemental Table S5). Interestingly, when comparing larger datasets of 40 SPD samples, 80 SPD, and 120 SPD, we found a large overlap between the methods, with only 475 proteins being unique to the 40 SPD runs (Fig. 5*C*). These results suggest that comparisons between clusters of single cells might be similar when analyzed by 40 SPD and 120 SPD. However, we show that many of the lowly-expressed proteins that are found robustly in 40 SPD runs are only sporadically found in 80 and 120 SPD methods. As a result, robust clustering might not be feasible in these cases.

In the next step, we wished to examine the impact of distinct timsTOF SCP acquisition methods. In the last year, several approaches have been developed to ensure maximal coverage of the ion cloud. DIA-PASEF standard method was optimized by the pydiAID tool (29). We compared this standard method to the newly introduced Slice-PASEF method. Slice-PASEF employs continuous slicing of the precursor ion space with fragmentation of all ions in each slice (24). We optimized the method and tested 1- m/z frame, 2-frames, and 4-frames per cycle. We found a marked improvement in protein identification using the Slice-PASEF method compared to DIA-PASEF, with only minor differences observed among the three Slice-PASEF options (Fig. 5*E*, Supplemental Table S5). Based on these results, we selected the 2-frame Slice-PASEF method for subsequent experiments.

#### Single-Cell Analysis of Tumor Macrophages

**Fig. 6:**
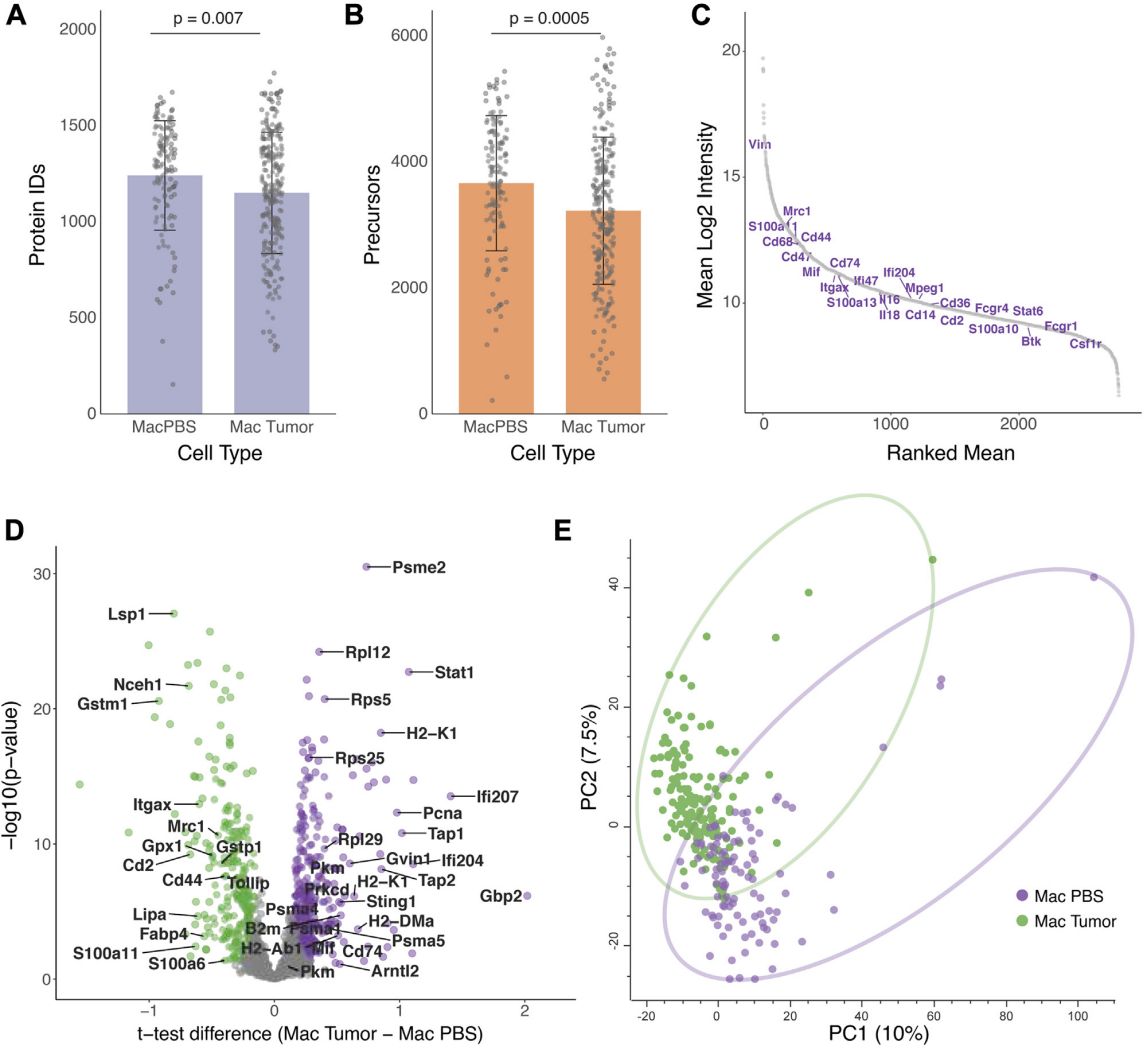
***In vivo* macrophage SCP.***A* and *B*, protein (*A*) and precursor (*B*) identification from lung macrophages from control lungs and tumor-bearing lungs (Mac Tumor N = 237; Mac PBS N = 126 single cells). *C*, an aggregated S-plot of the average macrophage proteins, identifies known macrophage markers. *D*, a volcano plot shows the significantly changing proteins between tumor and control macrophages. Two-sided T-tests were conducted with a permutation-based FDR cutoff of 5% and S_0_ = 0.1. The complete list of protein IDs is provided in Supplemental Table S6. *E*, principal component analysis shows unsupervised separation between tumor macrophages (*green*) and control macrophages (*purple*).

Moving from cultured cells to *in vivo* tumor cell populations poses additional challenges due to the smaller size of many cell types, particularly immune cell populations. We, therefore, investigated whether the optimized sample preparation protocol is sufficient to achieve biologically relevant information from immune cells in the tumor microenvironment. To that end, we injected MC38 murine colorectal cancer cells into the mouse tail vein to induce the development of lung metastases. After a week, we isolated the lungs and generated single-cell suspensions upon the appearance of small lesions. As a proof-of-concept, we focused our experiment on lung macrophages, sorted by flow cytometry and then dispensed as single cells into 1536-well plates on the cellenONE, as described above. Overall, we reached a maximal number of proteins of more than 1700 proteins and 6000 precursors in a single cell (Fig. 6, *A* and *B*, Supplemental Table S6). Examination of the identity of these proteins showed that we can identify the most known macrophage markers, including Cd68, Mrc1 (Cd206), Itgax (Cd11c), Fcgr1 (Cd64), Csf1r etc. (Fig. 6*C*). Furthermore, we were able to identify 575 significantly changing proteins between tumor macrophages and control lung macrophages (Fig. 6*D*, Supplemental Table S6). Specifically, we found that the tumor macrophages present increased expression of MHC molecules responsible for antigen presentation and multiple interferon-activated proteins (e.g., Ifi204, Ifi207, etc.). In agreement with the known anti-inflammatory role of Mrc1 (30), we found higher levels of Mrc1 in control macrophages than in tumor macrophages. These results show that with the current depth, we can capture the cellular activation state and associate these markers with hundreds of additional proteins in an unbiased way.

Finally, we examined whether the current analysis enables unbiased separation between macrophage proteomes. PCA analysis separated the cells into two main clusters, which match the separation between tumor macrophages and controls (Fig. 6*E*). Beyond the two main clusters, a few cells appeared as outliers. Analysis of the larger number of cells will suggest whether these can form additional smaller biologically relevant clusters. Overall, our results show a robust pipeline that enables the identification of important biological signals. We propose that this experimental approach can now be used for larger-scale SCP-based cancer research.

## Discussion

In this work, we describe a high-throughput sample preparation pipeline that enables a robust analysis of *in vivo* cells. The 1536-well format provides a readily available platform that is combined with semi-automated Evotip loading and LC-MS analysis on the Evosep1-timsTOF SCP using the Whisper Zoom 40 SPD method and Slice-PASEF. The coverage of ∼3000 proteins/cell is comparable to that of scRNAseq, but the low number of missing values is likely to simplify cell clustering and downstream statistical analyses. Notably, the experimental pipeline can also be combined with other instruments and is not limited to the Evosep-timsTOF setup.

The fixation of cells prior to single-cell dispensing offers dual advantages: it provides a practical stopping point in the workflow and serves a critical role in preserving the proteomic state of the cells. By halting cellular activity, fixation minimizes stress-induced proteomic alterations, thereby ensuring that the data accurately reflects the biological state of the cells at the time of collection. Our results are also supported by the recently published study showing similar advantages of cell fixation (31). This approach is especially valuable in proteomics studies with extended workflows, where prolonged handling can exacerbate cell stress, introducing variability and complicating the interpretation of results. For shorter experimental designs, where cells can be processed quickly, we recommend using non-fixed cells to achieve optimal proteomic coverage, reduce cell loss during washing steps, and minimize centrifugation. However, for longer experiments or high-throughput workflows, our findings indicate that mild fixation with 0.5% PFA for 15 min is preferable. This method allows cells to be stored at 4 °C overnight, providing an effective stopping point without compromising data quality. Further research is needed to evaluate the impact of extended storage durations.

The parallel processing of 1536 cells overcomes the first rate-limiting step of single-cell proteomics research. However, it transfers the limitation to the LC-MS acquisition step. We show that increasing the throughput to 120 SPD markedly reduces the number of identified proteins. Identification of ∼1500 proteins in HeLa cells using 120 SPD methods may be valuable. However, we speculate that the application of this method to *in vivo* immune cells might not reach sufficient depth. We acknowledge that this is a critical area for improvement that can be achieved with improved MS acquisition methods and instrumentation (e.g., Orbitrap Astral or timsTOF Ultra). Another approach to tackle the throughput challenge is to use a sample multiplexing method. For example, MS-level multiplexing by plexDIA (32) or dimethyl labeling (33) provide accurate MS-level quantification. However, they increase throughput by only 2-3-fold. In contrast, TMT labeling provides higher throughput but complicates downstream analyses due to potential TMT-batch effects and ratio compression (34, 35). While some of these limitations are resolved in the analyses of single TMT batches, the limited depth of single-cell analyses is expected to result in complicated normalization challenges between TMT sets. Of note, high multiplexing with TMT can only be achieved with very high resolution that is not yet reached on the timsTOF or the Astral analyzers (36).

Despite the limited LC-MS throughput, we show that the current state of single-cell proteomics already provides sufficient information to tackle biologically relevant questions in cancer biology and immunology. Tumor cells (cancer, stroma, and immune) can be used to unravel modes of cellular interactions and identify pathways related to cellular activation states. These studies are expected to provide important counterparts to the commonly used scRNAseq techniques. Ultimately, SCP will identify novel drug-resistance mechanisms and highlight potential cancer targets.

## Data Availability

All protein data is provided in supplementary tables included in the submission. All raw files and annotated spectra of single-peptide protein identifications are uploaded to PRoteomics IDEntifications Database (PRIDE). Data is available via ProteomeXchange Consortium with identifier **PXD058457**.

## Supplemental Data

This article contains supplemental data.

## Conflict of Interest

The authors declare that they have no conflicts of interest with the contents of this article.

## Supplemental Data

Supplemental Fig. S1 Proteomic changes of non-fixed HeLa single cells. STRING protein-protein interaction network of overnight (*A*) and same-day (*B*) non-fixed HeLa single cells. Nodes represent proteins, and edges indicate predicted or validated interactions based on evidence from the STRING database (version 12.0; confidence score >0.4). Highlighted proteins represent functionally related proteins revealing enriched pathways.
