# Supplementary figures and images for: High-Throughput Single-Cell Proteomics of In Vivo Cells
Source: Mol Cell Proteomics. 2025 Jun 20;24(7):101018. doi: 10.1016/j.mcpro.2025.101018 (PMC12301781; doi:10.1016/j.mcpro.2025.101018)

Supplementary Figure 1

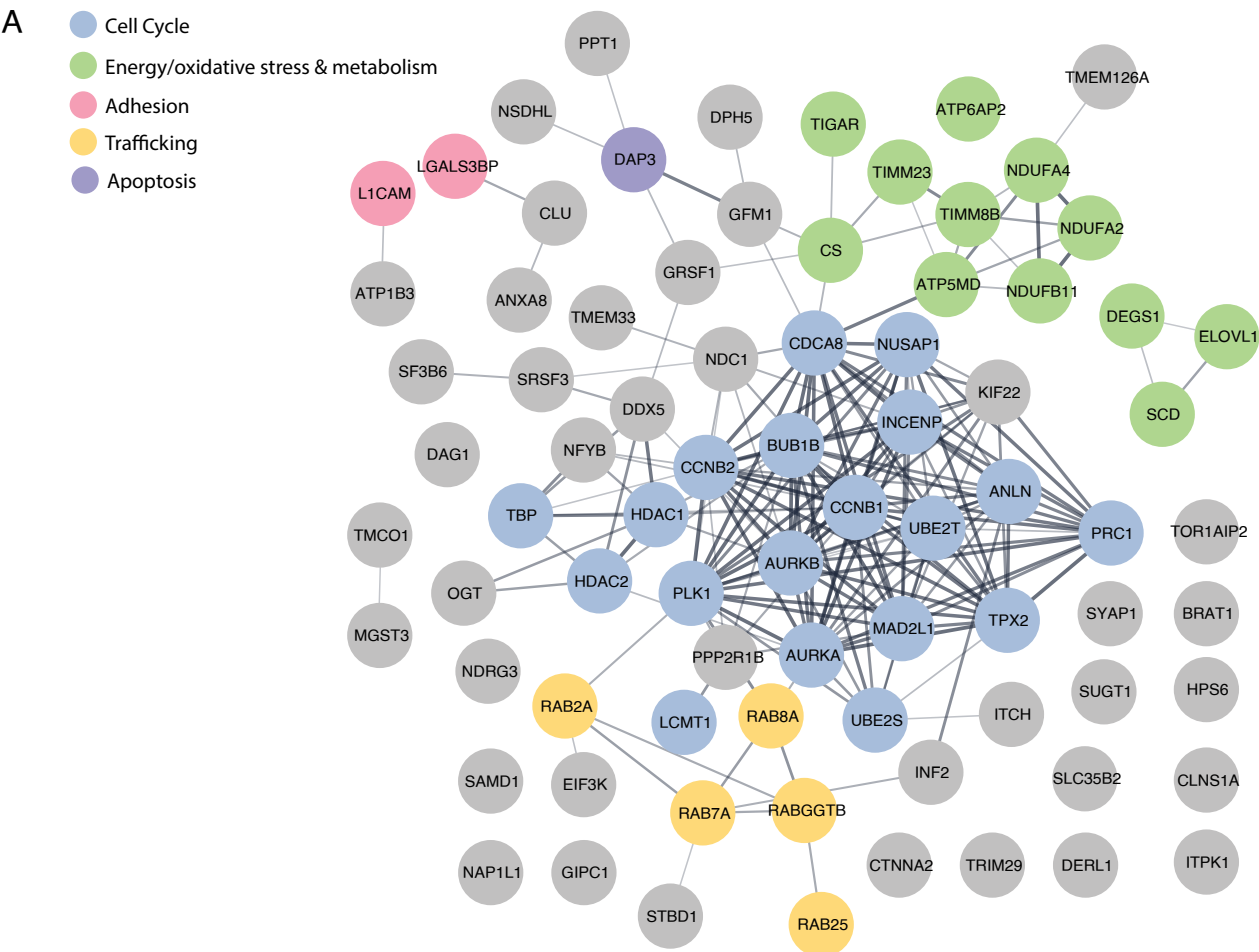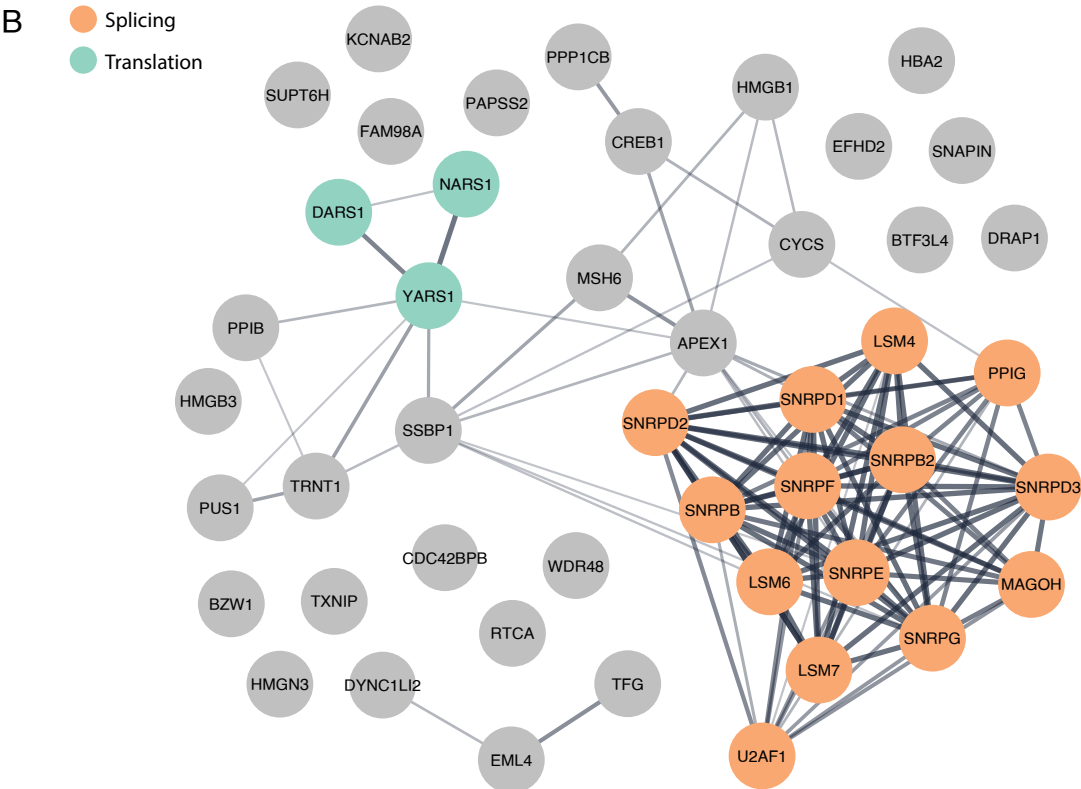

Supplement: Supplemental Fig. S1 — Proteomic changes of non-fixed HeLa single cells. STRING protein-protein interaction network of overnight (A) and same-day (B) non-fixed HeLa single cells. Nodes represent proteins, and edges indicate predicted or validated interactions based on evidence from the STRING database (version 12.0; confidence score >0.4). Highlighted proteins represent functionally related proteins revealing enriched pathways. [file mmc1.pdf]
